# Antioxidants and Exercise: A Redox-Informed Framework for Training Adaptation, Performance, and Recovery

**DOI:** 10.3390/antiox15040456

**Published:** 2026-04-07

**Authors:** Dan Cristian Mănescu, Andrei Tudor, Andreea Maria Mănescu, Iulius Radulian Mărgărit, Cătălin Octavian Mănescu, Ciprian Prisăcaru, Lucian Păun, Virgil Tudor

**Affiliations:** 1 Department of Physical Education and Sport, Bucharest University of Economic Studies, 010374 Bucharest, Romania; dan.manescu@defs.ase.ro (D.C.M.); andrei.tudor@eam.ase.ro (A.T.); manescuandreea19@stud.ase.ro (A.M.M.); iulius.margarit@defs.ase.ro (I.R.M.); catalin.manescu@defs.ase.ro (C.O.M.); 2 Faculty of Humanities, Valahia University of Târgoviște, 130105 Târgoviște, Romania; 3 Faculty of Physical Education and Sports, National University of Physical Education and Sports, 060057 Bucharest, Romania; virgil.tudor@unefs.ro

**Keywords:** exercise, antioxidants, hormesis, redox signaling, polyphenols, vitamins C and E, N-acetylcysteine, recovery, performance, training adaptation

## Abstract

Exercise-derived reactive oxygen species (ROS) are required for mitochondrial and hypertrophic adaptations, creating a practical trade-off: antioxidant strategies may support short-term performance and recovery yet blunt training signals when mis-timed or over-dosed. We performed a structured narrative review informed by transparent database searches of MEDLINE, Scopus, and SPORTDiscus (2000–2025), prioritizing human intervention studies and using mechanistic evidence to interpret plausibility. Evidence was mapped by antioxidant class, dose, timing, training modality, and context. Across trials, chronic high-dose vitamins C/E taken close to key sessions are most consistently associated with attenuation of redox-sensitive signaling, whereas food-first polyphenols and selected bioactives (e.g., tart cherry/anthocyanins, pomegranate, and curcumin) more often support recovery when positioned away from adaptation-critical workouts, without clear evidence of impaired training gains. N-acetylcysteine can acutely improve tolerance to repeated high-intensity exercise, but effects during prolonged training remain uncertain and appear context-dependent. We propose Redox-Adaptive Periodization, aligning antioxidant class, dose, and timing with the primary objective (adaptation vs. immediate readiness) and environmental constraints, and we outline methodological priorities to advance precision redox management.

## 1. Introduction

For decades, exercise-generated reactive oxygen species (ROS) were framed as unavoidable by-products of metabolism whose damage should be “neutralized” [1,2,3,4,5]. That view is now incomplete [6,7,8]. The same oxidants that can impair macromolecules also act as indispensable signals that initiate and amplify training adaptations—mitochondrial biogenesis, angiogenesis, remodeling of excitation–contraction coupling, and, in resistance training, hypertrophic signaling [9,10,11,12,13,14,15]. This dualism explains why antioxidant supplementation has produced conflicting findings: it can ease symptoms and preserve output in the short term, yet, when used indiscriminately or at high doses around training, it may dampen redox-sensitive pathways that athletes seek to strengthen [16,17,18,19,20,21,22,23,24].

Two additional forces sustain the controversy. First, “antioxidant” is a heterogeneous category. It encompasses direct radical scavengers (e.g., vitamins C and E), thiol donors and precursors (e.g., N-acetylcysteine), mitochondrial or membrane-localized compounds (e.g., CoQ10, alpha-lipoic acid), and diverse polyphenols that modulate signaling through Nrf2, AMPK, sirtuins, and inflammatory cascades [25,26]. These effects are often indirect—via redox-sensitive transcription and kinase networks—rather than simple radical “quenching” [27,28,29,30,31]. Second, methodology varies widely across trials. Biomarkers of oxidative stress range from robust (e.g., F_2_-isoprostanes) to notoriously labile (e.g., TBARS alone) [32,33,34,35,36]. Dosing, timing, background diet, training load, and environmental stress (heat, hypoxia, and pollution) are often insufficiently controlled. These sources of heterogeneity can hide genuine effects, create spurious ones, or invert conclusions when context shifts [16,17,18,19,20,21,22,23,24].

Redox biology offers a unifying lens. Most adaptations follow a hormetic pattern: a moderate perturbation in cellular redox state activates transcriptional programs and enzymatic defenses; too little signal, and adaptation is muted; too much, and damage and fatigue dominate. This “dose–response” also has a lower bound: overly reducing conditions (“reductive stress”) may be maladaptive, highlighting that redox balance—rather than maximal antioxidant capacity—is the operative target [8,26,36,37,38,39,40]. From this standpoint, the central practical question is not whether antioxidants are “good” or “bad,” but when they should be used, which class fits the goal, and how dose and timing interact with training so that protective effects do not erase the signal to adapt.

This review addresses that problem for athletes and practitioners seeking evidence-based guidance. We integrate mechanistic redox biology with human intervention studies and translate the resulting synthesis into a practical framework. We therefore focus on five interlinked questions:

**Q1**—How do exogenous antioxidants intersect with redox-sensitive pathways that underpin endurance and resistance adaptations?
**Q2**—How do different antioxidant classes affect acute performance and post-exercise recovery across modalities?
**Q3**—How do endogenous antioxidant systems adapt to training, and how does supplementation interact with those adaptations?
**Q4**—Which populations and contexts (training status, sex and age, dietary pattern and energy availability, heat or hypoxia, and congested competition schedules) shift the balance from benefit to risk?
**Q5**—Which methodological practices and biomarker panels best capture meaningful effects and should anchor future trials?

To answer these questions, we adopt a structured narrative review with transparent, reproducible methods. We prioritize human trials and use mechanistic work to interpret direction and plausibility. Evidence is organized by outcome domain—training adaptations versus acute performance/recovery—and analyzed through moderators that matter in real programs: class of antioxidant, dose, timing relative to key sessions, training phase, and environmental stress. Where meta-analysis is not appropriate, we apply structured synthesis principles and map the strengths and gaps in the literature.

Our contribution is twofold. First, we provide a clear taxonomy of antioxidant strategies—isolated vitamins, thiol donors, mitochondrial-targeted agents, and food-first polyphenols—positioned against their likely mechanisms and practical windows of use. Second, we propose Redox-Adaptive Periodization (RAP), a framework that matches type, dose, and timing to the goal (drive adaptation versus secure immediate availability/performance) and the context (e.g., heat, altitude, and congested fixtures). RAP reframes antioxidant use from blanket supplementation to signal management: preserve the exercise signal when adaptation is the priority; buffer selectively when performance or recovery in hostile environments is the constraint.

Finally, we identify methodological standards that can elevate the field: rigorous diet and load control, validated biomarker panels with appropriate sampling windows, and statistical approaches that accommodate non-linear dose–response relationships and inter-individual variability. Taken together, this agenda moves beyond the false dichotomy of “antioxidants: yes or no” toward precision redox management that respects the biology of hormesis while serving the realities of training and competition.

## 2. Methodological Approach: Evidence Sourcing and Synthesis

### 2.1. Review Design and Reporting

We conducted a structured narrative review to integrate mechanistic redox biology with human intervention evidence in exercise and sport [41]. The design emphasizes transparency and reproducibility while retaining the interpretive flexibility required for mechanism-informed synthesis. Because interventions, doses, timings, and outcomes are highly heterogeneous, we synthesized evidence narratively and used direction-of-effect coding (increase/decrease/no clear change/mixed [context-dependent]) supported by structured evidence tables rather than meta-analysis [42]. The objective was interpretive integration of heterogeneous evidence rather than exhaustive capture of all eligible studies or a formal systematic review synthesis.

### 2.2. Scope and Eligibility

The primary focus was human intervention evidence in healthy participants (≥16 years), spanning from recreationally active individuals to trained athletes of any sex. Eligible interventions included single-compound supplements and food-based strategies with plausible antioxidant or redox-modulating actions (e.g., vitamins C/E, N-acetylcysteine, CoQ10, alpha-lipoic acid, and polyphenol-rich interventions such as tart cherry, pomegranate, cocoa flavanols, curcumin, and resveratrol). Outcomes were grouped a priori into two domains: (i) training adaptations (≥2 weeks of structured training; endurance and/or resistance outcomes) and (ii) acute performance and recovery (single- or few-session studies assessing performance within ≤24 h and/or recovery over ~24–72 h). Biomarkers were recorded to support interpretation but were not treated as primary endpoints unless paired with functional outcomes. English-language full texts published from January 2000 to the most recent search update (November 2025) were considered.

To further structure the eligibility criteria, a PICO framework was applied:

**Population (P):** healthy individuals aged ≥16 years, including recreationally active individuals and trained athletes;
**Intervention (I):** antioxidant supplementation and food-based antioxidant strategies (e.g., vitamins, polyphenols, N-acetylcysteine, and mitochondrial-targeted compounds);
**Comparator (C):** placebo, control conditions, or baseline (pre-intervention) measures;
**Outcomes (O):** training adaptations (e.g., mitochondrial and hypertrophic responses), acute performance, and recovery-related functional outcomes.

### 2.3. Information Sources and Search Strategy

We searched MEDLINE (via PubMed), Scopus, and SPORTDiscus from January 2000 to the most recent update (November 2025), restricting to human studies where filters were available. The January 2000 onward timeframe was selected to ensure relevance to contemporary training practices, analytical methods, and biomarker validity. Searches combined antioxidant classes/compounds and food-based interventions with exercise/training terms and outcome keywords (performance, recovery, and adaptation), and were adapted to each database’s syntax and controlled vocabulary. We complemented database searching with backward and forward citation chasing of eligible studies and relevant reviews, and used Web of Science Core Collection for targeted citation tracking. To improve capture of ongoing or unpublished trials, we screened ClinicalTrials.gov and ISRCTN. Full database-specific search strings, limits, and the update date are provided in Supplementary Table S1.

The January 2000–November 2025 window applied to the formal database search only. Foundational pre-2000 papers and a small number of highly relevant post-search references identified during citation chaining and final contextual updating were added manually and were not part of the primary search yield.

### 2.4. Study Selection, Synthesis, and Appraisal

Records were screened at the title/abstract and full-text stages using predefined eligibility criteria. Screening and study selection were conducted by multiple authors, with eligibility decisions discussed and resolved by consensus to ensure consistency and minimize selection bias. A piloted extraction framework captured population characteristics, training modality and load, intervention dose/timing/formulation, comparator details, environmental context (e.g., heat or altitude), and primary functional outcomes with their sampling windows. Internal validity was appraised pragmatically, drawing on RoB 2 for randomized trials and ROBINS-I for non-randomized interventions to contextualize confidence in direction-of-effect conclusions [43,44]. Evidence is organized by outcome domain and antioxidant class, with key studies summarized in the corresponding tables and interpreted through pre-specified moderators (training status, sex/age, diet/energy availability, and environmental stress). Full database-specific search strategies are provided in Supplementary Table S1, extraction fields and appraisal procedures are described in Supplementary Text S2, and risk-of-bias summaries are provided in Supplementary Table S2.

### 2.5. Review Positioning and Rationale

This study was conducted as a structured narrative review integrating mechanistic redox biology with human intervention evidence in exercise and sport. Although multiple antioxidant classes have been examined in human trials, substantial heterogeneity exists in intervention type, dose, and formulation, timing relative to exercise, training modality and duration, baseline dietary exposure, environmental context, and biomarker selection and sampling windows.

Given this variability, quantitative pooling of outcomes was not considered appropriate, as combining mechanistically and contextually diverse endpoints could obscure meaningful differences [45,46]. Instead, evidence was synthesized narratively and organized according to predefined outcome domains (training adaptations versus acute performance and recovery) and relevant moderators (antioxidant class, dose, timing, training phase, and environmental stress).

This review was not registered in PROSPERO because it does not aim to produce pooled quantitative estimates but rather to provide an integrated, mechanism-informed synthesis of heterogeneous intervention evidence. PRISMA-style flow reporting is provided in Supplementary Figure S1 for transparency of study identification and selection; however, the review does not claim methodological equivalence with a registered systematic review [47,48].

A protocol outlining eligibility criteria and outcome domains was drafted prior to screening. Any minor refinements are documented in Supplementary Text S1. All quantitative values reported in Tables were extracted directly from the cited literature; full search strategies are provided in Supplementary Table S1, and the extraction framework is provided in Supplementary Text S2.

## 3. Redox Signaling in Exercise: The Mechanistic Scaffold

### 3.1. Sources and Kinetics of Reactive Species During Exercise

Skeletal muscle generates reactive oxygen and nitrogen species (ROS/RNS) from multiple nodes: mitochondrial electron transport (complexes I/III), NADPH oxidases (notably NOX2 at the sarcolemma/T-tubules), xanthine oxidase (especially during high flow–reperfusion dynamics), nitric oxide synthases (nNOS, eNOS), and, in specific contexts, uncoupled reactions in the endothelium [49,50,51,52,53,54,55]. Hydrogen peroxide (H_2_O_2_) functions as a diffusible second messenger; superoxide (O_2_^•−^, the superoxide radical anion) is often the first ROS formed and is rapidly dismutated to H_2_O_2_ by superoxide dismutase (SOD) isoforms; peroxynitrite and lipid peroxyl radicals arise under higher flux states [50,56,57,58]. Importantly, redox signaling is compartmentalized—mitochondria, cytosol, nucleus, and intermyofibrillar versus subsarcolemmal regions can experience distinct redox microdomains [59].

The time course of redox signaling is layered. Exercise induces a rapid, transient rise in H_2_O_2_ and redox relay activity (e.g., peroxiredoxin oxidation) within minutes, followed by transcriptional responses peaking hours later and structural adaptations accruing over weeks [60,61,62]. Critically, the shape of the signal matters: brief, moderate perturbations are pro-adaptive, whereas prolonged or excessive perturbations are more likely to be damaging [56,63,64].

### 3.2. Redox-Sensitive Nodes That Couple Work to Adaptation

Key sensors and effectors include: AMPK/SIRT1/PGC-1α (mitochondrial biogenesis), p38 MAPK and CaMKII (activity-dependent signaling), NRF-1/2 (mitochondrial transcription), mTORC1 (protein synthesis and hypertrophy), Nrf2/Keap1 (cytoprotective gene induction), NF-κB (inflammation), and HIF-1α (hypoxic/angiogenic programming) [65,66,67,68,69,70]. Many of these are redox-tunable via cysteine switches, kinase/phosphatase balance, or altered NAD^+^/NADH ratios [56,71]. Training strengthens endogenous antioxidant capacity—SOD1/2, catalase, GPx, peroxiredoxins, thioredoxin, and glutathione (GSH)—reshaping the resting redox tone and improving resilience to subsequent bouts [49,50,57].

Redox signaling is a reversible cysteine-based information transfer—exercise-derived H_2_O_2_ functions less as a damaging oxidant and more as a spatially and temporally regulated second messenger encoded through reversible redox post-translational modifications (PTMs) [56,64,71]. Many redox-sensitive pathways are tuned via transient cysteine oxidation events—including sulfenylation, disulfide bond formation, and S-glutathionylation—which alter kinase/phosphatase balance, transcription factor activation, and protein–protein interactions [71,72]. In this framework, peroxiredoxins (Prx) and the thioredoxin (Trx) system operate not merely as antioxidant defenses but as relay nodes that transmit H_2_O_2_ signals to selective downstream targets while preserving compartmental specificity [61,73,74,75,76].

Implications for exercise adaptation—because these relays are transient, compartmentalized, and kinetically constrained, the magnitude and timing of oxidant exposure determine whether signaling is amplified, shaped, or prematurely terminated [56,61,63]. Adaptive remodeling, therefore, depends not simply on the presence of reactive species, but on preserving the fidelity of these reversible signaling events [71,73,74]. Interventions that indiscriminately flatten peak ROS may truncate relay-dependent activation of mitochondrial and anabolic pathways, whereas strategies that respect signal amplitude and timing are more likely to maintain hormetic adaptation [63,77].

These integrator nodes interact with calcium transients, mechanical load, substrate availability, and inflammatory cues [62]. This helps explain why antioxidant interventions can show modality-specific effects—redox modulation of AMPK–PGC-1α signaling is most relevant for endurance adaptation, whereas redox effects on mTORC1 and satellite cell programs matter more for resistance training [62,65,66,67,68,77]. In the synthesis, we therefore separate chronic adaptation outcomes from acute performance/recovery outcomes and interpret findings through a dose, timing, and training context (see tables).

### 3.3. Hormesis and the “Redox Window”

Adaptations follow a non-linear (U-shaped) curve with respect to oxidant burden. Too little ROS leads to weak signaling, whereas too much leads to macromolecular damage, fatigue, and impaired remodeling. Exogenous antioxidants can shift this curve [56,63,64,77]. Agents that blunt peak ROS at the wrong time or dose risk erasing the very signals (PGC-1α, Nrf2, satellite cell activity) that training relies on [62,66,67]. By contrast, indirect antioxidants (e.g., many polyphenols) often precondition via Nrf2 or mitochondrial stress-signaling rather than acting as simple scavengers [77,78,79,80].

Operationally, this creates two practical windows [63,77]. During adaptation-focused blocks, the priority is to preserve the transient, compartmentalized ROS pulse around key sessions (the “adaptation window”). During congested competition, travel, or extreme environments, the priority may shift toward selective buffering to protect readiness (the “buffering window”), ideally in short pulses and with agents less likely to flatten signaling [77,78]. The remainder of the review evaluates how different antioxidant classes shift athletes along this continuum.

### 3.4. Practical Implication

Mechanistic biology predicts context-sensitive effects [62,63]. When the goal is adaptation, preserve the signal: avoid high-dose, peri-exercise scavengers that flatten redox transients [77,78]. When the goal is availability/performance now (e.g., congested fixtures, extreme environments), targeted buffering and anti-inflammatory support may be advantageous [77,81]. These principles motivate the later RAP framework.

**Figure 1:**
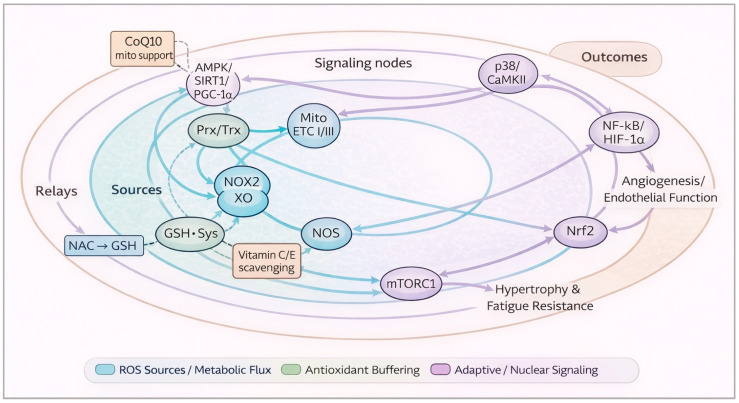
**Redox signaling topology in exercise.** Concentric ellipses depict the hierarchical organization of redox signaling from sources (mitochondrial ETC I/III, NOX2, xanthine oxidase, and NOS) [49,50,51,52,53,54,55], through relay systems (peroxiredoxin/thioredoxin and glutathione) [61,73,74,75,76], to signaling nodes (AMPK/SIRT1/PGC-1α, p38/CaMKII, Nrf2, mTORC1, and NF-κB/HIF-1α) [62,65,66,67,68,69,70] and phenotypic outcomes (mitochondrial capacity, angiogenesis/endothelial function, hypertrophy, and fatigue resistance). Curved connections represent conceptual information flow (line thickness indicates relative influence). Colors differentiate functional domains without implying quantitative differences. Antioxidant classes are positioned relative to the signaling architecture without disrupting adaptive redox signaling: CoQ10 (mitochondrial support) [82], N-acetylcysteine (glutathione support) [76,81], vitamins C/E (scavenging) [77,78], and polyphenols (Nrf2 support) [67,79,80]. The scheme emphasizes qualitative, compartmentalized redox signaling across mitochondrial, cytosolic, and nuclear domains [59] and is conceptual rather than quantitative.

To consolidate the mechanistic scaffold developed, Figure 1 maps the hierarchical flow from ROS sources to relay systems, downstream redox-sensitive nodes, and phenotypic outputs. It also visualizes the typical points at which different antioxidant classes interface with this signaling architecture.

Exercise evokes brief, compartmentalized ROS pulses that act as second messengers rather than mere by-products [56,59,63,64]. Two high-flux relays—Prx/Trx and GSH—transmit these signals to integrator nodes controlling metabolism and transcription [61,73,74,75,76]. Through AMPK/SIRT1/PGC-1α and p38/CaMKII, redox transients couple workload to mitochondrial biogenesis and metabolic flexibility; Nrf2 activates cytoprotective programs; NF-κB/HIF-1α and mTORC1 integrate inflammatory/hypoxic stress with the anabolic signal [62,65,66,67,68,69,70,71]. The peripheral phenotypes in the Figure represent exactly the outcomes we evaluate in the synthesis: mitochondrial capacity, endothelial function/angiogenesis, hypertrophy, and fatigue resilience.

The badges indicate antioxidant intervention points relative to the topology. Vitamins C/E operate as upstream scavengers relative to the relays—hence high peri-exercise doses can truncate the adaptive signal [77,78]. NAC (supports glutathione) can be selectively useful, in pulses, in contexts with high oxidative load (heat, altitude, and congestion), but requires individual testing [77,81]. Polyphenols (support Nrf2) support a food-first approach, distant from sessions; CoQ10 supports the ETC without dampening the ROS pulse [67,79,80,81,82]. This logic underpins, in the following sections, the complementary timing and interpretation of the various results.

## 4. Antioxidant Taxonomy and Practical Pharmacology

Because “antioxidant” is a functional label rather than a single pharmacological class, interpreting supplementation trials requires a mechanism-first taxonomy. In applied terms, the mechanism determines timing: compounds that act as upstream scavengers close to training can flatten the transient ROS pulse that initiates adaptation, whereas interventions that act indirectly (via transcriptional, vascular, or inflammatory modulation), or that are used as short pulses under defined constraints, are less likely to erase the training signal [83,84,85,86]. The taxonomy below is used throughout the review and provides the logic for how evidence is organized and operationalized in Redox-Adaptive Periodization.

### 4.1. Mechanistic Classes and Where They Interface with Redox Signaling

Direct scavengers—most notably vitamins C and E—can quench reactive species and interrupt lipid peroxidation chains, but they also operate “upstream” of key redox relays (e.g., glutathione and thioredoxin systems) [87]. This positioning helps explain why repeated, peri-exercise high-dose vitamin C/E has been linked to attenuation of mitochondrial and hypertrophic signaling in some training studies, even when acute soreness is reduced [2,9,10]. In practice, correcting deficiency or low dietary intake is appropriate; routine high-dose use immediately around key sessions is harder to justify when the primary objective is adaptation [85].

Thiol donors and redox buffers occupy a different niche. N-acetylcysteine (NAC) supports cysteine availability and glutathione (GSH) synthesis and can, in some athletes, improve tolerance to repeated high-intensity work or help preserve output under high oxidative load (e.g., heat, hypoxia, and congested schedules) [15,16,17]. At the same time, its effects are variable, dose-limited by gastrointestinal tolerance, and plausibly conditional on baseline redox tone—features that make it better suited to pre-tested, time-limited pulses than to continuous, ‘always-on’ supplementation across a mesocycle [17,88].

Route, exposure, and chronic use considerations—the interpretation of NAC evidence requires careful distinction between intravenous (IV) and oral administration. IV protocols provide mechanistic proof-of-concept under controlled laboratory conditions but have limited ecological validity for sport practice [88]. Oral NAC, in contrast, produces more variable systemic cysteine availability and is constrained by gastrointestinal tolerance and dose ceilings, resulting in pronounced inter-individual responder patterns [89]. While short loading phases may improve repeated high-intensity performance in selected contexts, evidence for chronic NAC use during prolonged adaptation blocks remains limited and inconclusive [17]. Accordingly, routine long-term supplementation cannot be recommended, and NAC is best conceptualized as a context-specific, pre-tested intervention when oxidative load becomes a limiting factor [17,88].

Other thiol-related or redox-cycling compounds (e.g., alpha-lipoic acid and oral glutathione) share mechanistic plausibility but currently lack the volume and consistency of athletic performance evidence observed with NAC [90,91,92].

Alpha-lipoic acid (ALA) and oral glutathione have plausible biochemical actions but comparatively limited athletic evidence; their practical role remains tentative and context-dependent [90,91,92].

Mitochondrial or membrane-localized agents (e.g., CoQ10 and mitochondria-targeted quinones such as MitoQ) are often grouped under the antioxidant umbrella, yet their primary relevance may be compartment-specific support of electron transport, redox cycling, or redox tone rather than global scavenging. This mechanistic distinction matters: compartmental targeting can, in theory, reduce ‘collateral’ dampening of cytosolic signaling, but it does not guarantee meaningful performance or adaptation gains [93,94]. Where used, these agents should be evaluated against sport-relevant endpoints and integrated into a broader nutrition and training plan rather than treated as a stand-alone solution.

### 4.2. Polyphenols and Other Indirect Redox Modulators

Polyphenol-rich foods and standardized extracts (anthocyanins/tart cherry, pomegranate polyphenols, cocoa flavanols, green tea catechins, curcumin, and resveratrol) are frequently described as “antioxidants,” yet in vivo they often act less as direct scavengers and more as signaling modulators [95,96]. Their metabolites can influence Nrf2-mediated cytoprotective programs, endothelial function (e.g., nitric oxide bioavailability), and inflammatory cascades (e.g., NF-κB), aligning with the more consistent pattern of recovery support observed in short, competition-oriented protocols [18,19,20,21,22,23,24,25,26,95,96,97,98,99]. This also explains why a food-first approach—achieving exposure through a dietary matrix rather than stacked high-dose isolates—tends to be the most defensible default during adaptation blocks [85,95].

From a programming standpoint, polyphenols fit naturally into periodized use: maintain habitual polyphenol intake through diet across the season, and deploy time-limited concentrates around unusually damaging blocks or congested fixtures when readiness and recovery are the binding constraints [18,19,20,21,22,23,24,25,26]. The main practical risk is inadvertent ‘megadose stacking’ (multiple concentrates plus high-dose vitamins), which can shift exposure from modulatory to non-physiological boluses with uncertain effects on signaling and gastrointestinal tolerance [85,100].

### 4.3. Practical Pharmacology: Formulation, Safety, and Compliance

Formulation and bioavailability materially shape exposure and, therefore, interpretation. Vitamin E requires co-ingestion with fat; many polyphenols undergo extensive phase II metabolism and microbiome transformation; and curcumin is a classic case where formulation (phytosome, micelle, and piperine co-administration) can change apparent bioavailability and interaction risk [87,95,101]. Consequently, dose–response should be interpreted in the context of the tested form, the dosing schedule, and the athlete’s background diet rather than the ingredient name alone.

Safety considerations are usually manageable but not trivial: NAC and large polyphenol boluses can provoke gastrointestinal symptoms; high-dose vitamin E may interact with anticoagulants; and melatonin should be treated primarily as a chronobiotic with timing-dependent effects on sleep and next-day alertness [87,88,95]. For competitive athletes, supplement quality control and anti-doping risk should be explicitly managed (e.g., third-party-tested products), and choices should be revisited whenever medications, travel, or training constraints change [100,101,102,103,104].

Table 1 provides a compact reference for the major classes, typical trial doses, common timing patterns, and practice notes. This pharmacology-first lens is used throughout the synthesis to keep mechanistic plausibility, dosing realism, and timing aligned with the outcomes that matter in training and competition.

**Table 1:** Antioxidant classes, mechanisms, typical trial doses, and practice notes.

Class/ Compound	Primary Action(s)	Typical Trial Dose/Form	Timing Patterns Studied	Bio Availability Notes	Common AEs	Interaction Flags	Practice Note
**Vitamin C**	Aqueous radical scavenger; regenerates E	500–1000 mg/day; 0.5–1 g pre/post	Chronic near training; acute pre-exercise	SVCT-mediated, saturable	GI upset at high doses	Enhances non-heme iron absorption; high-dose use may increase kidney-stone risk	Avoid chronic peri-exercise in adaptation blocks unless correcting deficiency
**Vitamin E** **(α-tocopherol)**	Lipid chain-breaking scavenger	200–800 IU/day	Chronic	Requires fat for absorption	GI, rare bleeding risk at high doses	Anticoagulants	Similar caution as vitamin C; avoid high doses peri-exercise
**NAC**	Cys donor that supports glutathione (GSH); direct scavenging	600–1200 mg acute; short courses 2–7 d	60–90 min pre-exercise; short performance blocks	Oral bioavailability moderate; IV research-only	GI upset, sulfur taste	Caution with nitroglycerin (headache/hypotension)	Consider for repeated-bout performance or heat/hypoxia; trial in training
**CoQ10** **(ubiquinone/** **ubiquinol)**	ETC carrier; membrane antioxidant	100–300 mg/day	Chronic	Better with fat; ubiquinol increases exposure	GI	Warfarin interaction reported	Mixed effects; safe/neutral in athletes
**ALA**	Mitochondrial redox cycling	150–600 mg/day	Chronic	R-ALA more active	GI, hypo-glycemia in sensitive	Diabetes meds	Sport evidence limited; small RCT suggests improved recovery during intensive microcycles; long-term training effects unclear.
**Anthocyanin** **-rich foods** **(tart cherry,** **berries)**	Nrf2 activation; anti-inflammatory; sleep support (tart cherry)	Juice/concentrate; 2 × 30–60 mL/day or equivalent	3–10 d pre-competition and through competition/recovery	Food matrix matters; microbiome metabolism	Rare GI	Minimal	Useful for DOMS/recovery in competition weeks
**Pomegranate** **polyphenols**	Endothelial/Anti-inflammatory	250–500 mL juice or equivalent extract	2–7 d and acute pre-exercise	Polyphenol metabolites drive effects	GI in large volumes	None major	May aid blood flow in endurance events
**Cocoa** **flavanols**	NO bio-availability; endothelial	300–600 mg CF/day	1–7 d and acute	Processed chocolate varies widely	Caffeine in chocolate	None major	Consider for submax/endurance support
**Curcumin** **(phytosome)**	NF-κB modulation; soreness	500–1000 mg/day (std extract); lower if phytosome	1–3 d pre-damaging work and 2–3 d post-damaging work	Low oral bioavailability; phytosome increases	GI; piperine interactions	Anti-coagulants	Recovery support; avoid peri-strength if concerned about signaling (data limited)
**Resveratrol**	SIRT1/AMPK activator	150–500 mg/day	Chronic	Low bioavailability	GI, headaches	CYP interactions	Potential to blunt adaptations in older men at high doses—use cautiously
**Astaxanthin**	Membrane antioxidant	4–12 mg/day	Chronic	Lipid carriers aid	Well tolerated	—	Neutral/mixed for performance
**Melatonin**	Indirect antioxidant; sleep	1–5 mg pre-sleep	Nights before competition/travel	Chronobiotic; timing critical	Next-day sleepiness	Sedatives	Use for jet-lag/sleep with circadian guidance

**Notes:** Dose ranges and timing patterns are representative of protocols used in human exercise studies and reviews discussed in this article. References are provided per antioxidant class corresponding to each row of the Table (vitamins C/E: [18,20,21,105,106,107,108,109,110]; N-acetylcysteine: [88,111,112,113]; mitochondrial/membrane-localized agents: [82,91,114]; polyphenol/bioactive strategies: [98,101,115,116,117,118,119,120,121,122,123]; astaxanthin/melatonin: [124,125,126]). These values reflect commonly used protocols rather than prescriptive recommendations. Bioavailability, adverse-event, and interaction flags are included for practitioner awareness and should be interpreted alongside current clinical guidance.

## 5. Effects on Training Adaptations (Chronic Outcomes)

### 5.1. Endurance Adaptations

Across endurance-training RCTs (2–16 weeks), high-dose vitamin C/E near training sessions has been repeatedly associated with attenuated upregulation of mitochondrial biogenesis markers (e.g., PGC-1α expression, downstream targets) and smaller gains in oxidative enzyme activity in some studies, with others reporting neutral effects [105,106,107]. Heterogeneity stems from dose, training status, and timing. The direction of effect leans toward risk of blunting when these vitamins are used chronically and peri-exercise.

For polyphenols, chronic intake during endurance training generally shows neutral-to-supportive signals for performance surrogates (e.g., time-to-exhaustion, submaximal efficiency) and no consistent evidence of blunted mitochondrial signaling when timing is away from key sessions. Resveratrol represents a context-specific exception. Some trials—primarily in older men—have reported attenuated training responses, potentially reflecting interactions within AMPK–SIRT1 signaling networks that alter angiogenic or mitochondrial remodeling under certain dosing conditions. However, these findings derive from relatively small samples and specific populations characterized by distinct baseline vascular and redox profiles. Accordingly, extrapolation to young, well-trained athletes should be cautious. Rather than constituting a blanket contraindication, current evidence suggests prudence with sustained high-dose resveratrol during adaptation-focused training blocks until dose–timing–training interactions are clarified in larger trials [127].

CoQ10 and ALA have not consistently enhanced training adaptations; the balance of evidence is neutral with occasional benefits in special populations (statin users, chronic fatigue), which lie outside pure athletic cohorts [114,128,129].

A structured synthesis of chronic antioxidant interventions during endurance training is presented in Table 2.

**Table 2:** Chronic antioxidant interventions during endurance training (≥2 weeks) and direction of effect on mitochondrial and performance adaptations.

Antioxidant (Class)	Population (Status; n) [Ref]	Dose & Timing	Training Protocol (Duration; Modality)	Primary Adaptation Outcomes	Direction	Key Moderators	RoB
**Vitamin C** **(direct scavenger)**	Trained men (*n* = 14) [105]	1 g/day; chronic; peri-exercise	8 wk endurance training	Mitochondrial biogenesis markers; endurance performance	Decrease mitochondrial markers; performance: mixed	High dose; proximity to sessions	Some concerns
**Vitamins C/E (direct scavengers)**	Young men & women (*n* = 54) [106]	1000 mg C + 235 mg E/day; chronic	11 wk endurance training	Cellular adaptation markers; VO_2_max	Decrease cellular adaptation; VO_2_max: no clear change/mixed	Sex; training status	Low
**Vitamins C/E**	Healthy adults [107]	Chronic supplementation during training	Exercise training intervention	Metabolic/health adaptations	Attenuate exercise-induced metabolic benefits	Baseline metabolic status	Low
**Resveratrol** **(polyphenol bioactive)**	Aged men (*n* = 27) [127]	Chronic; during training	Exercise training program	VO_2_max; vascular adaptations	Attenuate training response	Older age; AMPK/SIR 1 modulation	Low
**MitoQ** **(mitochondria-targeted)**	Untrained middle-aged men [128]	20 mg/day; chronic	3 wk HIIT	VO_2_peak; peak power; CS activity	VO_2_peak: no clear change; peak power: increase	Short intervention; untrained	Some concerns
**NAC** **(thiol donor)**	Recreational men [129]	Acute responses pre/post training period	6 wk Sprint-interval training	Redox markers; fatigue physiology	Context-dependent (training modifies NAC effect)	Training status; redox baseline	Some concerns
**CoQ10** **(mitochondrial support)**	Mixed training status [114]	100–300 mg/day; chronic	Various endurance programs	Performance endpoints	No clear change/mixed	Dose hetero-geneity	N/A

Notes: Direction summarizes the overall direction of training-related adaptation outcomes (mitochondrial biogenesis markers, oxidative enzymes, VO_2_max, or performance endpoints) as reported in the primary study, using the descriptors increase, decrease, no clear change, or mixed (context-dependent). When signaling and functional outcomes diverged, both are indicated. “Peri-exercise” denotes supplementation administered within the hours surrounding key training sessions. Risk of bias (RoB) reflects RoB 2 appraisal for randomized intervention trials; reviews were not formally appraised. HIIT = high-intensity interval training; CS = citrate synthase. Where the sample size was not reported, or the row summarizes evidence from reviews, n is not specified.

Table 2 illustrates a consistent pattern: chronic high-dose direct scavengers (vitamins C and E), particularly when administered near training sessions, are associated with attenuation of mitochondrial or cellular adaptation markers, although effects on whole-body performance outcomes are more variable. In contrast, mitochondria-targeted or redox-modulating compounds show largely neutral effects on core endurance adaptations within the durations studied. These findings support the principle that timing and mechanistic class, rather than the generic label “antioxidant,” determine the impact on training adaptation.

### 5.2. Resistance/Hypertrophy

Resistance-training studies with vitamin C/E provide mixed results. Some report smaller increases in hypertrophy-related signaling (p70S6K phosphorylation) or lean mass accretion; others show no difference in 1RM strength. When effects emerge, they trend toward attenuation of hypertrophic signaling with high-dose, peri-exercise vitamin C/E [108,109,110]. Evidence with polyphenols is sparse but largely neutral regarding hypertrophy; proprietary tea-extract blends appear largely neutral for strength gains, whereas curcumin formulations are better supported for post-exercise soreness/recovery than for enhancement of hypertrophic adaptation [130].

A structured summary of resistance-training interventions and hypertrophy-related outcomes is provided in Table 3.

**Table 3:** Antioxidant supplementation during resistance training and direction of effect on hypertrophic and strength adaptations (≥6 weeks).

Antioxidant (Class)	Population (Status; n) [Ref]	Dose & Timing	Training Protocol (Duration; Modality)	Primary Adaptation Outcomes	Direction	Key Moderators	RoB
**Vitamins** **C/E** **(direct** **scavengers)**	Young men & women (*n* = 54) [108]	1000 mg C + 235 mg E/day; chronic; peri-exercise likely	10–12 wk heavy-load resistance training	p70S6K signaling; lean mass; maximal strength	Decrease signaling; hypertrophy: no clear change; strength: mixed	High-dose; training status; timing proximity	Low
**Vitamins** **C/E** **(direct** **scavengers)**	Elderly men, 60–81 y (*n* = 34) [109]	500 mg C + 117.5 mg E before & after training (per session); 12 wk	12 wk strength training (3 sessions/week)	Lean mass (DXA); muscle thickness (US); 1RM strength	Decrease lean-mass gains; strength gains: no clear change	Older age; peri-exercise high-dose	Some concerns
**Vitamin** **C/E** **(direct** **scavengers)**	Healthy men trained & untrained; (*n* = 28) [110]	Vitamin C 1 g/day + vitamin E 400 IU/day for 11 wk (started 4 wk before pre-testing); taken before breakfast	Eccentric training: 4 wk (2×/wk) + acute eccentric bouts pre- and post-training	Muscle performance; redox biomarkers; hemolysis; muscle damage indices	No clear change	Training status; eccentric model; biomarker selection	Some concerns
**Polyphenol blend** **(tea extracts; indirect** **antioxidant)**	Untrained men (*n* = 40) [130]	2000 mg/day proprietary polyphenol blend; 4-wk preload; continued daily during training	4 wk supplementation + 6 wk progressive full-body RT (3 d/wk)	Lower-body 1RM strength; systemic TAC/oxidative markers	Strength gains: no clear change; TAC: increase	Proprietary blend (tea extracts + caffeine); untrained status; industry funding	Some concerns
**Vitamins** **C/E** **(direct** **scavengers)**	Trained men (*n* = 23) [131]	1000 mg C + 235 mg E/day; mornings; 10 wk	10 wk RT + ~300 kcal/day surplus (hypertrophy-oriented)	Fat-free mass (DXA); upper/lower body strength; VAT	Upper-body hypertrophy/strength: small decrease; most outcomes: no clear change; VAT gain: decrease	Energy surplus context; trained status; peri-session timing	Some concerns

Notes: Direction summarizes resistance-training adaptations, including acute anabolic signaling (e.g., p70S6K phosphorylation), hypertrophy-related outcomes (lean mass or cross-sectional area), and maximal strength (1RM), using the descriptors increase, decrease, no clear change, or mixed (context-dependent). When acute signaling and long-term morphological outcomes diverged, both are indicated. “Peri-exercise” denotes supplementation administered within the hours surrounding resistance sessions. Risk of bias (RoB) reflects RoB 2 appraisal for randomized intervention trials; reviews were not formally appraised.

Table 3 indicates that high-dose direct scavengers (vitamins C and E) may attenuate acute anabolic signaling responses to resistance exercise, yet consistent impairment of long-term hypertrophy or strength gains has not been demonstrated. Polyphenol-based strategies, particularly when distanced from key sessions, appear largely neutral with respect to hypertrophic adaptation while potentially supporting recovery. Resistance-induced morphological adaptations, therefore, seem relatively robust, although caution with chronic peri-exercise high-dose scavenger use remains justified. The same logic likely extends to concurrent programs: avoid high-dose scavengers around strength-priority sessions, whereas food-first polyphenols placed away from key workouts appear compatible with both modalities.

Taken together, the chronic training literature suggests a pattern that is more coherent than the headline controversy implies. When high-dose vitamins C and E are used repeatedly and, in particular, close to training sessions, several trials report attenuation of mitochondrial or hypertrophy-related signaling, although null findings also occur and likely reflect meaningful differences in dose, timing, training status, and outcome measurement [108,109,110,131]. In contrast, food-first polyphenol strategies, especially when placed away from key sessions, appear largely compatible with adaptation and may confer ancillary benefits related to vascular function or perceived recovery. For CoQ10, alpha-lipoic acid, and astaxanthin, the balance of evidence in athletic cohorts is mostly neutral [114,128,129]. Important uncertainties remain—notably whether repeated N-acetylcysteine exposure during prolonged training influences adaptation (positively, negatively, or not at all), and whether mitochondria-targeted compounds translate mechanistic promise into meaningful training outcomes.

## 6. Effects on Acute Performance and Recovery

### 6.1. Performance Within a Session or Within 24 h

Acute performance effects are most plausible when oxidative load contributes meaningfully to fatigue within the session. N-acetylcysteine (NAC) shows one of the clearest short-term signals: in repeated high-intensity or prolonged submaximal protocols, single doses or short loading courses can improve tolerance and, in some settings, performance—consistent with improved thiol availability and glutathione-dependent buffering [111,112,113]. However, effects are not universal, and both gastrointestinal tolerance and individual responsiveness should be tested in training well before competition use.

Direct scavengers (vitamins C and E) rarely improve acute performance in adequately nourished athletes and are difficult to justify outside of deficiency correction [78]. By contrast, selected polyphenol-rich strategies can yield small, context-dependent benefits, most often through vascular and perceptual pathways (e.g., cocoa flavanols or pomegranate in protocols where oxygen delivery is limiting) and, in congested schedules, through improved next-day readiness when sleep is supported.

### 6.2. Recovery over 24–72 h

Anthocyanin-rich interventions (tart cherry, berries) frequently reduce DOMS and preserve muscle function at 24–72 h after damaging exercise without clear evidence of impaired adaptations when used transiently around competitions. Pomegranate polyphenols and curcumin demonstrate similar recovery-support signals in eccentric/DOMS models. Sleep-supportive effects of tart cherry may further aid recovery via circadian/sleep pathways.

A structured synthesis of acute antioxidant interventions and short-term performance and recovery outcomes is presented in Table 4.

**Table 4:** Acute antioxidant strategies and short-term effects on performance and recovery (≤24 h performance; 24–72 h recovery).

Antioxidant (Class)	Population (Status; n) [Ref]	Dose & Timing	Protocol Type	Primary Functional Outcome	Direction	Context Sensitivity	RoB
**NAC** **(thiol donor)**	Trained men [111]	IV infusion pre-exercise	Prolonged submax cycling	Time to fatigue	Time to fatigue: increase	Laboratory setting; IV protocol	Some concerns
**NAC** **(oral)**	Well-trained triathletes (*n* = 10; 8 completed) [112]	1200 mg/day orally for 9 days (loading)	Cycle ergometer race simulation during intense training	Sprint performance during race simulation	Performance: increase	Small sample; GI tolerance; inter-individual variability	Some concerns
**NAC** **(systematic** **review)**	Adult males (healthy/active/athletes; 16 trials) [113]	Acute to short-term; doses varied	Controlled trials across multiple performance models	Performance + laboratory biomarkers (redox, GSH, etc.)	Mixed; overall trend toward performance improvement	High heterogeneity; baseline redox status	Variable/assessed
**Alpha-lipoic acid** **(ALA)**	Resistance- and Endurance-experienced men (crossover; *n* = 17) [91]	Single: 150 mg immediately post-session; Short-term: 300 mg/day (150 mg 2 h pre + 150 mg immediately post) during 6 d intensified training	Acute + short-term intensified training	Back squat performance; muscle damage (CK, myoglobin); inflammation (IL-6); soreness	Recovery: improve (modest)	Timing (pre/post); training load; baseline redox status	Some concerns
**Tart** **cherry** **(anthocyanins)**	Endurance runners [115]	~8 days preloading	Marathon/long-distance run	Muscle soreness	Recovery: improve (reduced soreness)	Running-induced damage	Low
**Tart** **cherry** **concentrate**	Semi- professional soccer (*n* = 16) [116]	30 mL × 2/day; 8 days	Intermittent sprint activity	Recovery markers; performance proxies	Recovery: improve	Congested match schedule	Low
**Tart** **cherry** **powder** **(Montmorency; anthocyanins)**	Endurance-trained runners/triathletes (men & women; *n* = 27) [117]	480 mg/day for ~10 d (7 d pre + day-of + 48 h post endurance challenge)	Endurance running challenge	Performance + recovery biomarkers (catabolism, inflammation, redox balance); soreness	Performance: increase; stress markers: decrease	Short-term pre-loading; formulation; training status	Some concerns
**Pomegranate juice**	Resistance-trained men (*n* = 17) [118]	250 mL twice/day for 15 days + 250 mL immediately post-exercise	Eccentric damage model (elbow flexors + knee extensors)	Isometric strength recovery; muscle soreness	Recovery: improve (elbow flexors); knee extensors: no clear change	Upper vs lower limb effects	Some concerns
**Pomegranate juice**	Elite Weightlifters [119]	750 mL/day for 48 h pre + 500 mL 60 min pre-session	Weightlifting training session (cross-over vs placebo)	Soreness; RPE; muscle-damage/inflammation biomarkers	Recovery: improve	High muscle-damage load; short pre-load	Some concerns
**Pomegranate** **extract**	Active Adults [120]	Acute or short-term	Endurance/time-trial models	Performance outcomes	No clear change/slight increase	Vascular modulation; dose	Some concerns
**Curcumin** **(CurcuWIN^®^; pre-loading)**	Physically active men & women (*n* = 63) [102]	CurcuWIN^®^ daily for 8 wk: 250 mg (50 mg curcuminoids) or 1000 mg (200 mg curcuminoids)	Downhill running (muscle-damaging)	Isokinetic torque/power; soreness (1–72 h post)	Recovery: improve (200 mg); no clear change (50 mg)	Dose-dependent; model (eccentric running)	Some concerns
**Curcumin** **(bioavailable)**	Physically active adults [121]	Pre- and post-exercise	EIMD protocol	DOMS; functional recovery	Soreness: decrease; performance: mixed	Formulation; timing	Some concerns
**Turmeric** **formulation**	Active men [122]	Acute dosing	DOMS model	Muscle pain; CK	Soreness: decrease	Bio-availability	Some concerns
**Cocoa** **flavanols** **(systematic** **review)**	Mixed populations & modalities [123]	Various doses/ durations; heterogeneous	Multiple Exercise models (performance & recovery)	Performance, vascular and oxidative stress outcomes (varies by study)	Mixed	Dose/composition; co-ingestants (e.g., caffeine); training status	N/A (review)

Notes: Direction summarizes short-term functional outcomes, including time to fatigue, repeated-sprint ability, time-trial performance, muscle soreness (DOMS), strength recovery, and functional capacity at 24–72 h, using the descriptors increase, decrease, no clear change, or mixed (context-dependent). Where vascular or biomarker changes occurred without a clear performance benefit, both are indicated. “Multi-day” denotes short loading protocols (typically 3–10 days). Risk of bias (RoB) reflects RoB 2 appraisal for randomized intervention trials; reviews were not formally appraised.

Table 4 demonstrates a clearer signal for recovery-oriented interventions than for acute performance enhancement. N-acetylcysteine shows the most consistent benefits in repeated high-intensity or fatigue-sensitive protocols, although effects are variable and responder-dependent. Polyphenol-rich strategies, particularly anthocyanin- and pomegranate-based interventions, more consistently support recovery following muscle-damaging exercise without strong evidence of acute performance enhancement. These patterns reinforce the distinction between buffering fatigue in constrained contexts and preserving redox signaling during adaptation phases.

### 6.3. Timing Relative to Exercise: The Redox Window

The acute literature reviewed above suggests that the effects of antioxidant supplementation are strongly timing-dependent relative to the exercise stimulus. Exercise generates a rapid ROS burst that participates in redox-sensitive signaling during the minutes and early hours after the session; within this adaptive window, indiscriminate high-dose scavenging may attenuate molecular events linked to mitochondrial biogenesis, metabolic remodeling, and training adaptation. By contrast, later phases of recovery appear more permissive for targeted antioxidant support, particularly when the goal is to reduce soreness, preserve muscle function, or maintain readiness across repeated efforts [22,23,105,106,107,108,109,110,111,112,113,115,116,117].

**Figure 2:**
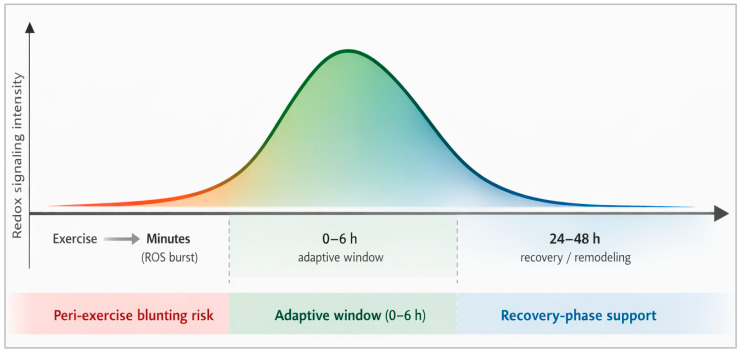
**Redox window vs. antioxidant timing.** Exercise induces a transient increase in reactive oxygen species (ROS) that functions as a signaling stimulus for molecular adaptation. The schematic depicts an early peri-exercise blunting-risk zone, a 0–6 h adaptive window, and a later 24–48 h recovery/remodeling phase. High-dose antioxidant exposure close to exercise may interfere with redox-sensitive signaling relevant to adaptation, whereas later recovery phases may provide a more appropriate context for targeted antioxidant support aimed at repair and readiness [22,23,105,106,107,108,109,110,111,112,113,115,116,117]. The scheme is conceptual and intended to guide timing interpretation rather than prescribe doses or specific compounds.

This timing framework helps reconcile apparently conflicting findings in the literature. Interventions that may be counterproductive when placed close to key adaptation-focused sessions can become useful when recovery is the immediate priority, especially in competition-dense schedules or under elevated physiological stress. Figure 2 summarizes this concept as a temporal redox window spanning the acute signaling phase and the later recovery/remodeling phase, emphasizing that antioxidant effects depend not only on class and dose, but also on when the exposure occurs relative to exercise [22,23,111,112,113,115,116,117].

### 6.4. Stressful Environments

Environmental stressors (heat, hypoxia/altitude, and high pollution exposure) can amplify oxidative and inflammatory load, increase perceived exertion, and compress recovery, conditions in which selective buffering may shift from ‘optional’ to ‘useful’. However, these stressors can also be part of the intended adaptive stimulus (e.g., altitude camps), so interventions should be periodized rather than applied chronically. The most plausible use-case is short, time-limited support during constrained weeks (travel, congested fixtures, and extreme heat), preferentially with strategies that have been pre-tested for tolerance and individual benefit (e.g., NAC in repeated-bout protocols, or polyphenol-rich interventions used primarily for recovery) [132,133,134,135].

In acute settings, the clearest performance signal emerges for N-acetylcysteine in protocols that tax repeated high-intensity capacity, consistent with a role for thiol availability and glutathione-dependent buffering; effects are less consistent for single-bout time trials. In contrast, isolated vitamins C and E seldom improve acute performance in adequately nourished athletes and are difficult to justify outside of deficiency correction. Polyphenol-rich foods and extracts show more consistent value for recovery: short, time-limited anthocyanin-rich protocols (e.g., tart cherry or berries) and selected bioactives (pomegranate polyphenols, curcumin) often reduce DOMS and help preserve muscle function over 24–72 h after damaging exercise, with benefits that may be amplified when sleep loss, travel, heat, or altitude increase overall stress [111,112,113,115,116,117,118,119,120,121,122].

## 7. Moderators and Personalization

### 7.1. Athlete Biology: Training Status, Sex, and Age

The same antioxidant exposure can have different consequences depending on baseline training status. Untrained or newly trained individuals typically exhibit larger redox perturbations and greater plasticity; in this context, chronic or peri-exercise high-dose scavengers are more likely to blunt redox-sensitive signaling that underpins early gains. In contrast, trained and elite athletes often show smaller redox excursions at a given absolute workload and may benefit from selective buffering in tightly constrained contexts (e.g., tournament weeks), provided that timing is chosen to avoid flattening the signal from the highest-priority sessions [23,105,106,107,112,116,127,128,129].

Sex and age further modify both redox tone and the balance between adaptation and recovery. Older adults may present with higher oxidative and inflammatory burden and sometimes show stronger recovery responses to polyphenol-rich strategies, yet they may also be more susceptible to attenuation of training responses with certain high-dose bioactives (e.g., resveratrol in older men). Female-specific evidence remains comparatively sparse; future trials should routinely document menstrual phase, hormonal contraceptive use, and iron status, as each can influence oxidative markers and recovery trajectories [127,136,137].

### 7.2. Nutritional Context: Baseline Diet and Energy Availability

Background diet is not a nuisance variable—it is part of the intervention context. When habitual fruit, vegetable, and polyphenol intake is adequate, isolated high-dose supplementation has less physiological justification and greater potential to behave as a pharmacological bolus. Conversely, correcting deficiency (e.g., low vitamin C status) is appropriate and should be treated as nutritional repletion rather than as a performance strategy. Wherever possible, food-first exposures should be the default during adaptation blocks, reserving concentrates for short windows with a clear rationale [85,100,136].

Energy availability is a particularly important moderator. Low energy availability can amplify oxidative and inflammatory stress, compromise immune function, and increase the perceived need for ‘recovery aids.’ However, using chronic high-dose scavengers as a substitute for adequate fueling is unlikely to resolve the underlying constraint and may still interfere with signaling. In practice, if low energy availability is suspected, the first-line intervention is nutritional and scheduling support; antioxidant strategies—if used—should be conservative, time-limited, and paired with monitoring of recovery and training tolerance [136,138,139].

### 7.3. External Constraints: Environment, Schedule, and Sleep Disruption

Heat, hypoxia/altitude, air pollution, congested fixtures, travel, and sleep disruption can all shift the benefit–risk balance toward targeted buffering because the limiting factor becomes immediate availability rather than long-term remodeling. In these settings, short pulses of selected strategies (e.g., NAC in proven responders; polyphenol-rich or anthocyanin-focused interventions used primarily for recovery) may help preserve readiness, but the supplementation window should not outlast the stressor window. This framing aligns with Redox-Adaptive Periodization: preserve signaling in build phases, and permit selective buffering only during brief periods when environment or schedule makes recovery the binding constraint [132,133,134,135,140].

Taken together, these moderators explain why the literature often looks inconsistent at first glance: the same compound can help in a constrained performance week yet hinder signaling when used chronically around adaptation sessions. Probable genotype-, metabolotype-, and microbiome-related responder patterns may contribute, but evidence is not yet strong enough to support prescribing on that basis; for now, these factors belong primarily to the future-research agenda. They also motivate the methodological emphasis of the next section—validated biomarkers, appropriate sampling windows, and reporting standards that allow future trials to stratify effects by context rather than averaging away meaningful conditional responses.

## 8. Biomarkers and Methodology—Measuring What Matters

### 8.1. Biomarker Quality Matrix

Oxidative stress biomarkers are a major driver of between-study inconsistency in the antioxidant–exercise literature. Several widely used assays are non-specific (e.g., TBARS/MDA without validated methods), highly sensitive to handling, or poorly aligned with the biology of compartmentalized redox signaling. To reduce interpretive errors, biomarker selection should prioritize validated measures of oxidative damage and redox couples (e.g., F_2_-isoprostanes, 8-oxo-dG, protein carbonyls, and GSH/GSSG), paired with functional outcomes and clear sampling windows [32,33,34,35,36,37,38,141].

A recurrent source of confusion is the conflation of oxidative damage with redox signaling. Damage markers—such as lipid peroxidation products, oxidized nucleotides, or protein carbonyls—reflect cumulative oxidative modification and are influenced by systemic spillover and sampling conditions. In contrast, adaptive signaling depends on transient, reversible redox events within defined cellular compartments, often mediated through cysteine-based modifications and relay systems (e.g., peroxiredoxin/thioredoxin networks). These signaling processes may not be captured by global “total antioxidant capacity” indices. Accordingly, biomarker panels should be selected to match the biological question—distinguishing between damage resolution, inflammatory modulation, and adaptive redox signaling [7,8,9,10,11,12,32,141].

Table 5 summarizes a practical quality matrix: preferred measures, common pitfalls, and minimal timing/processing standards. The central principle is alignment—biomarkers should match the question (damage vs signaling vs adaptation), the tissue of interest (systemic vs. muscle-local), and the time course of the response (minutes vs. hours vs. 24–72 h).

**Table 5:** Biomarker quality matrix: recommended measures, common pitfalls, and interpretation notes.

Domain	Preferred Measures (Higher Validity)	Use with Caution/Pitfalls	Timing & Interpretation Notes
**Lipid peroxidation**	F_2_-isoprostanes (plasma/urine)	TBARS alone; MDA without validated methods	Urine integrates over hours; pair with sampling windows and hydration control.
**DNA oxidation**	8-oxo-dG (urine/plasma); comet assay with oxidative enzymes	Single time points without baseline; assay drift	Interpret with baseline and workload; muscle-local events may not appear in blood.
**Protein oxidation/nitration**	Protein carbonyls; nitrotyrosine (with validated immunoassays)	Non-specific antibody panels; lack of standards	Best paired with functional outcomes; avoid over-interpreting small deltas.
**Redox couples & thiol status**	GSH/GSSG ratio; cysteine/cystine; oxidized/reduced peroxiredoxin states	Hemolysis; delayed processing; unreported storage	Requires strict processing and rapid quenching; biopsies improve specificity.
**Antioxidant capacity/enzymes**	SOD, GPx, catalase activity (standardized); targeted redox proteomics (if available)	TAC/FRAP as sole endpoint; non-standard units	Enzyme activity adapts with training; interpret as adaptation markers, not damage.
**Damage & inflammation** **(EIMD context)**	CK + myoglobin (with responder handling); IL-6/IL-10 panels; soreness + function tests	CK alone (high variability); untimed cytokines	Align with 24–72 h recovery window; report training load and prior activity.

**Notes:** Entries indicate relative methodological confidence rather than a universal hierarchy. “Preferred” identifies assays with stronger analytical specificity and clearer biological interpretability, whereas “caution” identifies measures with lower specificity, greater pre-analytical sensitivity, or weaker linkage to localized redox signaling. Results should therefore be interpreted in relation to sample matrix, timing, and handling, and global antioxidant-capacity indices should not be used alone [7,8,9,10,11,12,32,33,34,35,36,37,38,60,61,73,74,75,76,141].

### 8.2. Sampling Windows and Tissues

Sampling timing dictates interpretation. Immediate post-exercise values largely reflect acute flux and redox relay activation; sampling at ~3–6 h is more informative for early transcriptional programming; and 24–72 h windows better capture muscle damage, inflammatory resolution, and remodeling. When studies aim to infer training adaptation, at least one assessment beyond the acute window (e.g., next-morning signaling or enzyme activity) is preferable to single post-bout snapshots [32,38,141].

Whole-blood or plasma markers are practical but may miss muscle-local redox events; when biopsies are feasible, pairing tissue assays with standardized systemic markers improves mechanistic specificity. At minimum, studies should report pre-analytical handling (processing time, temperature, and freeze–thaw cycles) because these factors can dominate biomarker variance and obscure true intervention effects [32,141].

### 8.3. Training-Load and Diet Control

Training load should be quantified and reported (e.g., session-RPE × duration, TRIMP, and power/GPS-derived metrics) because redox and recovery responses scale with both intensity and accumulated stress. Between-group differences in load can masquerade as supplement effects if not measured and reported explicitly. Environmental stressors (heat, altitude) and recovery co-interventions (cold-water immersion, compression, and sleep strategies) should be documented for the same reason [132,133,134,135,140,142,143].

Dietary intake should be documented or standardized in the 24–48 h before key testing sessions, with explicit reporting of baseline antioxidant and polyphenol intake, energy availability, and concurrent ergogenic aids (caffeine, nitrates) and medications (NSAIDs). Whenever feasible, protocols should include a brief ‘supplement washout’ period and clear instructions to avoid stacking additional high-antioxidant products, so that observed effects can be attributed to the intervention rather than background variability [100,136,138].

### 8.4. Statistics for Non-Linear Responses

Given hormesis and strong inter-individual variability, non-linear and responder-aware statistics are often more appropriate than simple group mean comparisons. Where sample sizes permit, restricted cubic splines (dose–response), Bayesian hierarchical models (individual slopes), and measurement-error thresholds for ‘responders’ can clarify conditional effects. Trials should report clinically meaningful change thresholds (MCIDs) alongside *p*-values, and pre-specify how baseline redox tone will be handled (e.g., stratification or covariate adjustment) [144,145].

Future trials should predefine redox phenotypes (low vs high oxidative responders) and incorporate baseline stratification. Without accounting for baseline redox tone, intervention effects risk appearing inconsistent when they are in fact conditional.

A practical biomarker selection guide is summarized in Table 5, whereas a minimum reporting checklist for future trials is provided in Supplementary Table S3.

## 9. A Redox-Informed Framework for Antioxidant Use in Exercise

Training adaptation and day-to-day performance are shaped not only by workload, recovery, and environmental conditions, but also by how exogenous antioxidants interact with endogenous redox signaling. Because these interactions can either reinforce or interfere with adaptive remodeling, their effects cannot be treated as uniform across training phases or stressor profiles. A redox-informed approach, therefore, requires that antioxidant use be aligned with the intended training outcome, the physiological state of the athlete, and the temporal dynamics of exercise-induced oxidative signals.

**Figure 3:**
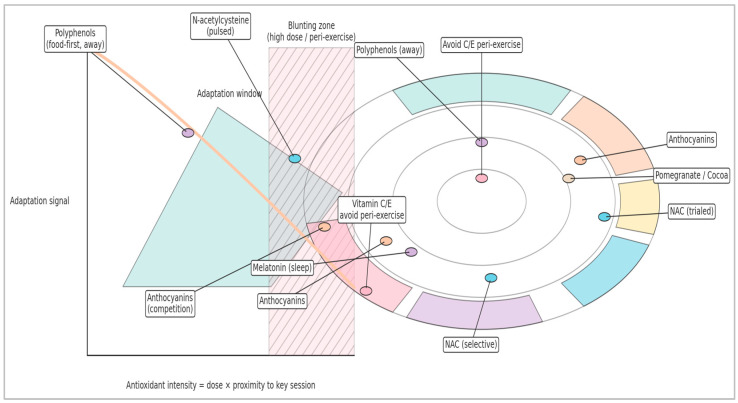
**Redox-adaptive periodization of antioxidant use in exercise**. On the left, the adaptation signal rises when exercise-generated ROS are permitted to engage canonical pathways (AMPK/SIRT1/PGC-1α, p38/CaMKII, Nrf2) within an adaptation window (mint), and is attenuated in a blunting zone when high-dose scavengers are taken near key sessions (rose, hatched). Colored pins mark class anchors; explanatory boxes indicate recommended positioning: polyphenols (food-first, away from training), N-acetylcysteine (pulsed/needs prior trial), anthocyanins (competition-oriented support), and vitamin C/E (avoid peri-exercise). On the right, the RAP compass (Redox-Adaptive Periodization) organizes context-specific decisions across phases (Build, Taper) and constraints (Heat, Altitude, Travel, and Congestion): preserve redox signaling in build, then permit targeted pulses (e.g., anthocyanins, polyphenol-rich juices) when availability or performance is paramount; reserve NAC for athletes who have pre-tested tolerance/benefit; use melatonin to consolidate sleep when travel or schedule congestion threatens recovery. Abbreviations: ROS, reactive oxygen species; RAP, redox-adaptive periodization; and NAC, N-acetylcysteine [22,23,85,95,100,104,105,106,107,108,109,110,111,112,113,115,116,117,126,132,133,134,135,140]. This Figure presents a conceptual, hypothesis-generating framework intended to guide timing and class selection rather than prescribe doses; recommendations should be individualized to training goals, context, and tolerance. The framework integrates both evidence-supported elements and theoretical assumptions, which are not equally supported by human intervention data.

Figure 3 illustrates this integrative logic, situating antioxidant strategies within a coherent, context-dependent decision structure. The schematic synthesizes goal orientation, contextual stressors, and timing considerations into a practical framework for applied use, and should be interpreted in light of the heterogeneous and context-dependent nature of the available evidence.

Figure 3 translates the hormetic view of exercise redox biology into actionable rules for practice. The central principle is that transient ROS elevations during and after exercise are part of the signal that drives adaptation, so antioxidant placement should be judged primarily by training objective and timing, not by a generic assumption that more buffering is always better [22,23].

In build phases, the default is conservative: prioritize food-first intake and avoid routine peri-exercise high-dose scavengers that may flatten redox-sensitive signaling. As the priority shifts toward readiness—during taper, competition congestion, heat, altitude, or travel—time-limited pulses of selected strategies can be justified, especially when they have been pre-tested for tolerance and individual benefit [100,132,133,134,135,136,137,138,139,140].

Thus, RAP is a placement framework rather than a dosing doctrine: it aims to preserve the biology that makes training effective while allowing selective support when performance, environment, or schedule becomes the dominant constraint.

### 9.1. Principles

Redox-Adaptive Periodization (RAP) treats antioxidant exposure as a training variable rather than a default daily habit. The guiding premise is goal primacy: strategies that protect short-term availability can be appropriate when the immediate constraint is performance, but they should not become routine in phases where the objective is adaptation, because the exercise-induced redox signal is itself a driver of remodeling [22,23].

In build phases, the practical implication is conservative: prioritize a food-first dietary pattern, and minimize routine, high-dose direct scavengers close to key sessions so that redox-sensitive signaling is not flattened. When the dominant constraint shifts—dense competition schedules, heat, hypoxia, travel, sleep disruption, or unusually high muscle-damage loads—selective buffering can be justified, ideally in time-limited pulses and only after tolerance has been established. Across all contexts, timing matters: distancing concentrated antioxidant boluses from the hours surrounding key workouts helps preserve training signals while still allowing targeted support for recovery and readiness [132,133,134,135,136,137,138,139,140].

These principles reflect the current balance of mechanistic and intervention evidence, which remains heterogeneous, and should be interpreted as context-sensitive guidance rather than fixed prescriptive rules.

### 9.2. Practical Implementation

Operationally, RAP begins by defining whether the immediate objective is adaptation or availability, then maps the constraints most likely to magnify oxidative and inflammatory load (e.g., heat, hypoxia, travel, sleep disruption, low energy availability, or unusually high muscle-damage exposure). Strategy selection follows from that logic: during build phases, routine high-dose vitamin C/E near key sessions is generally avoided, habitual polyphenol-rich foods can be maintained, and concentrated extracts are best reserved for clearly justified windows [22,23,100,132,133,134,135,136,137,138,139,140]. Accordingly, these strategies should be interpreted as guidance rather than prescriptive recommendations, given the heterogeneity of the available evidence.

During performance-focused blocks, short, time-limited pulses—such as anthocyanin-rich recovery support or, in proven responders, acute NAC in repeated-sprint or hostile-environment settings—can be used selectively, with monitoring of recovery, muscle function, sleep, and adverse effects [132,133,134,135,136,137,138,139,140]. A practical week-level illustration is provided in Supplementary Box S1.

A class-by-context summary of antioxidant positioning within RAP is provided in Table 6.

**Table 6:** Redox-Adaptive Periodization (RAP): Context-based positioning of antioxidant strategies relative to training objectives and environmental constraints.

Phase/ Constraint	Vitamins C/E (Scavengers)	NAC (Thiol Donor)	Polyphenols (Food-First)	Anthocyanins (Tart Cherry/Berries)	Pome-Granate	Curcumin	MitoQ/CoQ10	Mela-Tonin
**Build** **(adaptation** **priority)**	Avoid peri; avoid chronic high-dose	Rare	Food-first only	Not routine	Food-first	If needed, away from sessions	Likely neutral; limited data	Sleep only
**Intensified block**	Avoid peri	Consider short pulse	Moderate intake	Optional	Optional	If high muscle damage	Unclear	If sleep disturbed
**Taper/Competition week**	Avoid high-dose	Trial first; pulsed if responder	Moderate	Pulse (3–10 d)	Pulse	Short peri-damage window	Individual	If travel/late matches
**Heat/Hypoxia/Altitude**	Avoid chronic	Consider pulsed	Supportive	Useful	Supportive	Supportive	Uncertain	—
**Congested fixtures/Travel**	Avoid peri	If proven responder	Yes	Yes (recovery focus)	Yes	Yes (DOMS-heavy)	Optional	Yes
**Low energy availability**	Avoid chronic	Cautious	Food-first emphasis	Supportive	Supportive	Conservative	Neutral	Monitor sleep

Notes: RAP emphasizes antioxidant positioning relative to the biological objective of the training phase. “Avoid peri” indicates avoidance within the hours surrounding key sessions to preserve redox-sensitive signaling. “Pulse” denotes short-term use in performance- or recovery-constrained contexts. Recommendations reflect current evidence synthesis and are not prescriptive dosing guidelines [22,23,85,95,100,104,105,106,107,108,109,110,111,112,113,114,115,116,117,118,119,120,121,122,126,127,128,129,132,133,134,135,136,137,138,139,140].

Table 6, therefore, treats antioxidant exposure as a modifiable training variable: preserve signaling during build phases and reserve selective buffering for constrained performance contexts.

## 10. Gaps and Priorities for Future Research

Although the Redox-Adaptive Periodization (RAP) framework proposes a conceptual structure for aligning antioxidant use with training objectives and environmental constraints, several important knowledge gaps remain. Addressing these limitations will be essential to translate redox-informed strategies into precise and context-sensitive recommendations for athletes and practitioners.

Future work should prioritize designs that can disentangle dose from timing, ideally through factorial trials that independently manipulate each variable relative to exercise. Studies also need to become more sex- and hormone-aware, including careful documentation of menstrual phase, contraceptive status, and age-related physiology. Perhaps most importantly, outcome selection should move beyond laboratory proxies to sport-relevant endpoints—match performance, skill execution under fatigue, and validated recovery metrics—so that mechanistic findings can be interpreted in an applied context.

Environmental stressors (heat, altitude, and pollution) should be integrated into ecologically valid protocols, and mechanistic endpoints (e.g., redox proteomics, mitochondrial function assays) should be paired with functional outcomes. Given pronounced inter-individual variability, responder profiling using genotype, metabolotype, and microbiome features—supported by N-of-1 and Bayesian adaptive approaches—may be necessary to reach ‘precision’ recommendations. Finally, long-term safety, medication interactions, and direct comparisons between whole-food strategies and standardized extracts remain under-studied and should be treated as priorities rather than afterthoughts.

The restriction of the primary search to studies published from 2000 onward may have excluded earlier relevant findings; however, this decision was made to prioritize methodological consistency and applicability to contemporary research and practice.

## 11. Conclusions and Key Messages

Exercise-generated ROS are not enemies to be eliminated—they are indispensable signals. Exogenous antioxidants are, therefore, tools whose value depends on class, dose, timing, and context. Chronic peri-exercise vitamin C/E risks blunting adaptation; food-first polyphenols and pulsed NAC can support recovery and availability during competition or environmental stress when used judiciously. The RAP framework operationalizes these principles so practitioners can preserve the signal when building and buffer when competing. Methodological rigor—validated biomarkers, timing control, and non-linear analytics—is essential to move the field toward precision redox management.

The central shift is conceptual: antioxidants are not defaults, but modulators of signal timing, and their intelligent use requires alignment with the biology of adaptation.

## Data Availability

No new data were created or analyzed in this study. Data sharing is not applicable to this article.

## Supplementary Materials

The following supporting information can be downloaded at: https://www.mdpi.com/article/10.3390/antiox15040456/s1, Table S1: Full database-specific search strategies, limits, and update date; Supplementary Text S2: Extraction framework and credibility appraisal; Table S2: Risk-of-bias appraisal summaries (RoB 2 and ROBINS-I); Figure S1: PRISMA-style search and selection workflow; Supplementary Text S1: Deviations from the internal screening/synthesis plan; Table S3: Minimum reporting checklist for antioxidant–exercise intervention trials; Box S1: Illustrative microcycle example for applying RAP. All references cited in the Supplementary Materials are included in the main reference list.
