# Supplementary material for: Antioxidants and Exercise: A Redox-Informed Framework for Training Adaptation, Performance, and Recovery
Source: Antioxidants (Basel). 2026 Apr 7;15(4):456. doi: 10.3390/antiox15040456 (PMC13113188; doi:10.3390/antiox15040456)
Supplement: Supplementary file 1 [file antioxidants-15-00456-s001.zip › antioxidants-4223872-supplementary.pdf]

Supplementary Materials

Antioxidants and Exercise: A Redox-Informed Framework for Training Adaptation, Performance, and Recovery

Figure S1. PRISMA-style search and selection workflow

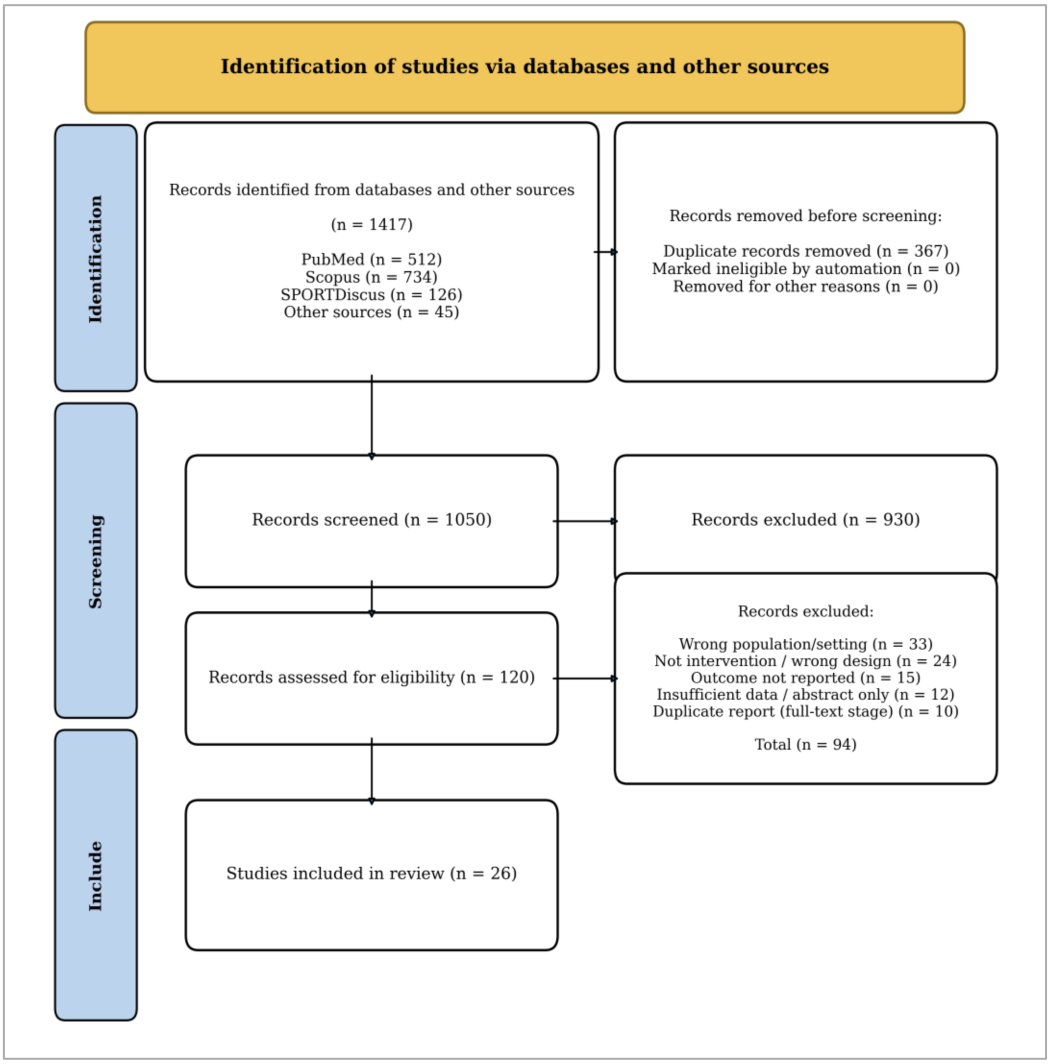

Table S1. Full database-specific search strategies, limits, and update date

Searches were run from January 2000 to November 2025. References outside this window were included only if added manually for foundational context or final contextual updating. Searches were restricted to humans where available, and limited to

English-language full texts. Syntax was adapted to each platform. The strings below reflect the final search queries executed in each database.

Database	Search strategy	Limits / notes
**MEDLINE (PubMed)**	("antioxidant*"[Title/Abstract] OR "oxidative stress"[Title/Abstract] OR "redox signaling"[Title/Abstract] OR "vitamin C"[Title/Abstract] OR "vitamin E"[Title/Abstract] OR polyphenol*[Title/Abstract] OR anthocyanin*[Title/Abstract] OR curcumin[Title/Abstract] OR pomegranate[Title/Abstract] OR "tart cherry"[Title/Abstract] OR "N-acetylcysteine"[Title/Abstract] OR NAC[Title/Abstract] OR "coenzyme Q10"[Title/Abstract] OR CoQ10[Title/Abstract] OR MitoQ[Title/Abstract] OR resveratrol[Title/Abstract] OR astaxanthin[Title/Abstract] OR "alpha lipoic acid"[Title/Abstract]) AND (exercise[Title/Abstract] OR training[Title/Abstract] OR "physical activity"[Title/Abstract] OR endurance[Title/Abstract] OR resistance[Title/Abstract] OR strength[Title/Abstract] OR sprint*[Title/Abstract] OR athlete*[Title/Abstract] OR sport*[Title/Abstract]) AND (performance[Title/Abstract] OR recovery[Title/Abstract] OR adaptation[Title/Abstract] OR hypertrophy[Title/Abstract] OR mitochondr*[Title/Abstract] OR fatigue[Title/Abstract] OR soreness[Title/Abstract])	Date window: January 2000 to November 2025; humans; English-language where filters were available.
**Scopus**	TITLE-ABS-KEY (antioxidant* OR "oxidative stress" OR "redox signaling" OR "vitamin C" OR "vitamin E" OR polyphenol* OR anthocyanin* OR curcumin OR pomegranate OR "tart cherry" OR "N-acetylcysteine" OR NAC OR "coenzyme Q10" OR CoQ10 OR MitoQ OR resveratrol OR astaxanthin OR "alpha lipoic acid") AND TITLE-ABS-KEY (exercise OR training OR "physical activity" OR endurance OR resistance OR strength OR sprint* OR athlete* OR sport*) AND TITLE-ABS-KEY (performance OR recovery OR adaptation OR hypertrophy OR mitochondr* OR fatigue OR soreness)	Date window: 2000–2025; document types prioritized original human research and relevant reviews for backward/forward citation chasing.
**SPORTDiscus**	TX (antioxidant* OR "oxidative stress" OR "redox signaling" OR "vitamin C" OR "vitamin E" OR polyphenol* OR anthocyanin* OR curcumin OR pomegranate OR "tart cherry" OR "N-acetylcysteine" OR NAC OR "coenzyme Q10" OR CoQ10 OR MitoQ OR resveratrol OR astaxanthin OR "alpha lipoic acid") AND TX (exercise OR training OR "physical activity" OR endurance OR resistance OR strength OR sprint* OR athlete* OR sport*) AND TX (performance OR recovery OR adaptation OR hypertrophy OR mitochondr* OR fatigue OR soreness)	Date window: 2000–2025; sports-specific indexing improved capture of applied athlete studies.

Table S2. Risk-of-bias appraisal summary

A. Study-level appraisal

Risk-of-bias judgments were used to contextualize confidence in direction-of-effect conclusions (not to compute pooled estimates). Randomized trials were appraised using RoB 2; non-randomized interventions used ROBINS-I. The table below provides the study-level overall judgment used in Tables 2–4 of the main manuscript.

Study	Design	Tool	Overall judgment
Gomez-Cabrera et al., 2008 (Vitamin C - direct scavenger)	Human intervention trial	RoB 2	Some concerns
Paulsen et al., 2014 (Vitamins C/E - direct scavengers)	Human intervention trial	RoB 2	Low
Ristow et al., 2009 (Vitamins C/E)	Human intervention trial	RoB 2	Low
Gliemann et al., 2013 (Resveratrol - polyphenol bioactive)	Human intervention trial	RoB 2	Low

Broome et al., 2022 (MitoQ - mitochondria-targeted)	Human intervention trial	RoB 2	Some concerns
Lemminger et al., 2023 (NAC - thiol donor)	Human intervention trial	RoB 2	Some concerns
Deng et al., 2025 (CoQ10 - mitochondrial support)	Systematic review/ meta-analysis	N/A	N/A (review; not formally appraised)
Isenmann et al., 2020 (Alpha-lipoic acid - ALA)	Human intervention trial	RoB 2	Some concerns
Paulsen et al., 2014 (Vitamins C/E - direct scavengers)	Human intervention trial	RoB 2	Low
Bjørnsen et al., 2016 (Vitamins C/E - direct scavengers)	Human intervention trial	RoB 2	Some concerns
Theodorou et al., 2011 (Vitamin C/E - direct scavengers)	Human intervention trial	ROBINS-I	Some concerns
Jäger et al., 2019 (Curcumin - CurcuWIN®; pre-loading)	Human intervention trial	RoB 2	Some concerns
Beyer et al., 2017 (Polyphenol blend - tea extracts; indirect antioxidant)	Human intervention trial	RoB 2	Some concerns
Martínez-Ferrán et al., 2023 (Vitamins C/E - direct scavengers)	Human intervention trial	RoB 2	Some concerns
Medved et al., 2004 (NAC - thiol donor)	Human intervention trial	RoB 2	Some concerns
Slattery et al., 2014 (NAC - oral)	Human intervention trial	RoB 2	Some concerns
Fernández-Lázaro et al., 2023 (NAC - systematic review)	Systematic review/ meta-analysis	N/A	N/A (review; not formally appraised)
Kuehl et al., 2010 (Tart cherry - anthocyanins)	Human intervention trial	RoB 2	Low
Bell et al., 2016 (Tart cherry - concentrate)	Human intervention trial	RoB 2	Low
Levers et al., 2016 (Tart cherry powder - Montmorency; anthocyanins)	Human intervention trial	RoB 2	Some concerns
Trombold et al., 2011 (Pomegranate juice)	Human intervention trial	RoB 2	Some concerns
Ammar et al., 2016 (Pomegranate juice)	Human intervention trial	ROBINS-I	Some concerns
Torregrosa-García et al., 2019 (Pomegranate extract)	Human intervention trial	RoB 2	Some concerns
Nicol et al., 2015 (Curcumin - bioavailable)	Human intervention trial	RoB 2	Some concerns
Amalraj et al., 2020	Human	RoB 2	Some

(Turmeric formulation)	intervention trial		concerns
Decroix et al., 2018 (Cocoa flavanols - systematic review)	Systematic review/ meta-analysis	N/A	N/A (review; not formally appraised)

B. Risk-of-bias appraisal domains used in synthesis

Summary of the recurrent appraisal issues encountered across antioxidant–exercise intervention studies and how they informed confidence in the synthesis.

Appraisal domain	Typical concern in included trials	Why it matters	Use in synthesis
**Randomization and allocation concealment**	Often underreported in small exercise trials; crossover washout not always justified.	Can exaggerate benefit estimates or make null effects hard to interpret.	Lowered confidence when direction of effect depended on one or two small trials.
**Blinding and placebo credibility**	Food-based interventions and strongly flavored supplements sometimes allow partial unblinding.	Expectation effects may influence soreness, RPE, and subjective recovery outcomes.	Objective outcomes were weighted more heavily when blinding was uncertain.
**Baseline diet and supplement control**	Habitual antioxidant intake, energy availability, caffeine, and additional supplements were inconsistently controlled.	Background intake can blunt or amplify apparent intervention effects.	Contextualized heterogeneity and limited strong claims about dose-response.
**Training-load standardization**	Session intensity, supervision, and external load quantification were variably reported.	Different training stimuli alter the redox challenge and adaptation signal.	Helped explain discordant findings across chronic training studies.
**Biomarker validity and timing**	Use of low-specificity markers or poorly aligned sampling windows remained common.	Mis-timed or weak biomarkers can disconnect measured oxidative stress from the biological question.	Biomarker findings were interpreted cautiously unless supported by validated markers and appropriate timing.
**Adherence, contamination, and co-interventions**	Pill counts/logs were common, but dietary contamination and recovery co-interventions were not always captured.	True exposure may differ from assigned exposure.	Reduced certainty for small acute studies reporting marginal benefits.
**Missing data and sample size**	Many studies were modest in size and not powered for interaction effects or responder analyses.	Raises imprecision and the chance of unstable estimates.	Favored direction-of-effect language over definitive magnitude claims.
**Selective reporting and protocol transparency**	Pre-specification of primary outcomes and accessible protocols were inconsistent.	Positive secondary outcomes may be overemphasized.	Findings were cross-checked against the full methods/outcome set when available.

Text S1. Deviations from the internal screening/synthesis plan

No substantive deviations were made to the inclusion criteria or outcome domains. Minor editorial updates were applied during manuscript polishing to improve internal consistency (e.g., alignment of table content with cited sources and completion of missing table numbering).

Text S2. Extraction framework and credibility appraisal

Extraction fields (piloted before full extraction) included: study design (parallel/crossover); population (training status, sex, age); intervention (compound/food, exact dose, formulation, timing relative to exercise, duration); comparator; training protocol and load quantification; environmental stress (heat/hypoxia/pollution); primary functional outcomes and timepoints; biomarker panel and sampling windows; adverse events and compliance; funding/conflicts.

Credibility appraisal was performed at the outcome level. Randomized trials were appraised using RoB 2 domains (randomization, deviations from intended interventions, missing outcome data, outcome measurement, and selective reporting). Non-randomized interventions were appraised using ROBINS-I domains (confounding, selection, classification of interventions, deviations, missing data, measurement, and reporting). For crossover designs, attention was paid to carry-over and period effects. Risk-of-bias judgments were used to contextualize confidence in direction-of-effect conclusions rather than to compute pooled estimates.

Table S3. Minimum reporting checklist for antioxidant–exercise intervention trials

This table corresponds to the reporting checklist cited from Section 8 of the main manuscript and was moved to Supplementary Materials to keep the main text focused on the synthesis.

Checklist item	Minimum to report
**Population**	Training status; sex/age; baseline diet pattern; baseline antioxidant intake (food + supplements); medication use.
**Intervention details**	Compound/class; exact dose (mg and mg/kg if relevant); formulation (e.g., R-ALA, phytosome); timing relative to exercise (pre/peri/post; distance in hours); duration.
**Comparator**	Placebo composition; blinding integrity; food control if food-based intervention.
**Compliance & adverse events**	Compliance method (pill count/log); GI symptoms; withdrawals and reasons.
**Training protocol**	Modality; intensity prescription; volume/progression; supervision; load quantification (e.g., sRPE×duration, TRIMP, GPS).
**Co-interventions**	Caffeine, nitrates, NSAIDs; sleep strategy; recovery modalities; instructions about additional supplements.
**Diet control**	Standardization window (e.g., 24–48 h pre-tests); energy availability; protein intake; key foods restricted/controlled.
**Environmental context**	Heat/hypoxia/pollution exposure; travel/jet lag; competition congestion.
**Outcomes**	Primary outcome(s) pre-specified; MCID/meaningful change thresholds; sport-relevant endpoints when possible.
**Biomarker panel**	Validated markers (e.g., F_2-isoprostanes, GSH/GSSG); sampling windows; tissue (blood vs biopsy); processing/storage.
**Statistics**	Non-linear or responder-aware approach (e.g., splines, Bayesian hierarchical); handling of baseline redox tone; missing data plan.

**Transparency**	Funding/conflicts; protocol availability; data/code availability statement; trial registration (if applicable).

Box S1. Illustrative microcycle example for applying RAP

This didactic week-level example corresponds to the practical implementation section of RAP cited in Section 9 of the main manuscript.

Box S1. Illustrative microcycle example for applying RAP (didactic; not prescriptive).

Scenario: team sport week with two matches (Tue and Thu), with the second match played in heat.

Objective: support availability and recovery during congestion without turning buffering into a chronic habit.

- Mon (training/activation): Maintain a food-first pattern (fruit/vegetables); avoid high-dose vitamins C/E and avoid NAC near the session.
- Tue (Match 1): Use anthocyanin-rich support in the evening after the match (e.g., tart cherry concentrate/juice) to support soreness and sleep; keep other concentrated antioxidants minimal.
- Wed (recovery/light session): Prioritize sleep and protein; if soreness is high, curcumin can be used later in the day, placed away from any quality session.
- Thu (Match 2, heat): For athletes with proven benefit and gastrointestinal tolerance, consider NAC (~600 mg, 60–90 min pre-match); avoid high-dose vitamins C/E peri-match; take anthocyanins in the evening post-match.
- Fri (recovery): Continue anthocyanins for 1–2 nights only if congestion persists; use melatonin only when travel/late kickoff disrupts circadian timing.
- Sat–Sun (return toward build): Discontinue concentrated pulses and revert to food-first intake; avoid routine high-dose scavengers around key sessions.
